# The Impact of Hurricane Sandy on HIV Testing Rates: An Interrupted Time Series Analysis, January 1, 2011‒December 31, 2013

**DOI:** 10.1371/currents.dis.ea09f9573dc292951b7eb0cf9f395003

**Published:** 2018-09-13

**Authors:** Linda I. Ekperi, Erin Thomas, Tanya Telfair LeBlanc, Erica Elaine Adams, Grete E. Wilt, Noelle-Angelique Molinari, Eric G. Carbone

**Affiliations:** Centers for Disease Control and Prevention, Office of Public Health Preparedness and Response, Division of State and Local Readiness, Applied Science and Evaluation Branch (ASEB), Atlanta, Georgia, USA; Centers for Disease Control and Prevention, Office of Public Health Preparedness and Response, Office of the Director, Office of Science and Public Health Practice (OSPHP), Atlanta, Georgia, USA; Centers for Disease Control and Prevention, Office of Public Health Preparedness and Response, Division of State and Local Readiness, Applied Science and Evaluation Branch (ASEB), Atlanta, Georgia, USA; Centers for Disease Control and Prevention, Office of the Director, Division of Toxicology and Human Health Sciences, Geospatial Research, Analysis, and Services Program (GRASP), Atlanta, Georgia, USA; Centers for Disease Control and Prevention, Office of the Director, Division of Toxicology and Human Health Sciences, Geospatial Research, Analysis, and Services Program (GRASP), Atlanta, Georgia, USA; Centers for Disease Control and Prevention, Office of Public Health Preparedness and Response, Division of State and Local Readiness, Applied Science and Evaluation Branch (ASEB), Atlanta, Georgia, USA; Centers for Disease Control and Prevention, Office of Public Health Preparedness and Response, Office of the Director, Office of Science and Public Health Practice (OSPHP), Atlanta, Georgia, USA

## Abstract

Background: Hurricane Sandy made landfall on the eastern coast of the United States on October 29, 2012 resulting in 117 deaths and 71.4 billion dollars in damage. Persons with undiagnosed HIV infection might experience delays in diagnosis testing, status confirmation, or access to care due to service disruption in storm-affected areas. The objective of this study is to describe the impact of Hurricane Sandy on HIV testing rates in affected areas and estimate the magnitude and duration of disruption in HIV testing associated with storm damage intensity.

Methods: Using MarketScan data from January 2011‒December 2013, this study examined weekly time series of HIV testing rates among privately insured enrollees not previously diagnosed with HIV; 95 weeks pre- and 58 weeks post-storm. Interrupted time series (ITS) analyses were estimated by storm impact rank (using FEMA’s Final Impact Rank mapped to Core Based Statistical Areas) to determine the extent that Hurricane Sandy affected weekly rates of HIV testing immediately and the duration of that effect after the storm.

Results: HIV testing rates declined significantly across storm impact rank areas. The mean decline in rates detected ranged between -5% (95% CI: -9.3, -1.5) in low impact areas and -24% (95% CI: -28.5, -18.9) in very high impact areas. We estimated at least 9,736 (95% CI: 7,540, 11,925) testing opportunities were missed among privately insured persons following Hurricane Sandy. Testing rates returned to baseline in low impact areas by 6 weeks post event (December 9, 2012); by 15 weeks post event (February 10, 2013) in moderate impact areas; and by 17 weeks after the event (February 24, 2013) in high and very high impact areas.

Conclusions: Hurricane Sandy resulted in a detectable and immediate decline in HIV testing rates across storm-affected areas. Greater storm damage was associated with greater magnitude and duration of testing disruption. Disruption of basic health services, like HIV testing and treatment, following large natural and man-made disasters is a public health concern.  Disruption in testing services availability for any length of time is detrimental to the efforts of the current HIV prevention model, where status confirmation is essential to control disease spread.

## Introduction

The devastation caused by Hurricane Sandy in late October of 2012 resulted in an estimated 117 deaths and over $70 billion in physical damage^1^^, ^2. Three hundred and seventy-five thousand housing units were destroyed in New Jersey and New York and 8.5 million people lost power across 21 states. More than 1,000 patients were evacuated from metro-area hospitals due to unsafe conditions such as untreated waste; 2.75 billion gallons flowed from treatment plants and contaminated the public water systems^3^^, ^4. The storm destroyed homes, displaced loved ones, and deprived many individuals of essential necessities. Vulnerable populations, or groups with medical or other functional and access needs, were disproportionally impacted and were particularly at risk for being exposed to post-disaster distress. Individuals infected with Human Immunodeficiency Virus (HIV) and their contacts are disproportionally affected during storm impacts as they lose access to testing and treatment due to disruptions in healthcare services.

The Centers for Disease Control and Prevention (CDC) recommends everyone ages 13 to 64 be tested for HIV at least once as part of routine health checks, and those with increased risk factors should get tested more frequently^5^. CDC also recommends healthcare providers test individuals who engage in high risk behaviors for acquiring and transmitting HIV^1^. However, the ability to follow recommendations and reach a local testing site might be impeded for persons who experience dislocation, damaged homes, and economic losses immediately after a disaster. The breakdown in security, damage to infrastructure, building loss and displacement of medical personnel associated with the hurricane could also result in inaccessibility of HIV testing and healthcare services. Disruptions in diagnostic testing, access to preventive measures like condoms and antiretroviral therapies, might be unavailable during major disasters contributing to increased risky behaviors and potential HIV transmission^6^. In addition, not knowing one’s status might contribute to the likelihood of spreading the disease through unsafe sex or progression of the disease to Acquired Immunodeficiency Syndrome (AIDS) and other associated opportunistic infections^5^.

According to the National Health Interview Survey, as of 2010, only 45% of people in the U.S. ages 18 to 64 were tested for HIV^7^. Treatment success requires early diagnosis, achievable through testing as it is estimated that 13% of the 1 million people living with HIV are unaware of their infection^7^. The goal of the National AIDS Strategy is to increase the percentage of people living with HIV who know their serostatus to at least 90 percent^8^^, ^9. Natural or man-made disasters that disrupt HIV testing and treatment can be a barrier to reaching the goal set by the National AIDS Strategy.

Our study examined the impact of Hurricane Sandy on HIV testing in affected states by Storm Impact Rank (SIR). We used Interrupted Time Series (ITS) analyses to estimate the impact of Hurricane Sandy on HIV testing rates among privately insured individuals not previously diagnosed with HIV. We estimated, the immediate impact as well as the duration of impact and used these estimates to calculate the number of missed testing opportunities attributable to the storm.

## Methods

**Study Population**

The study population was identified as privately insured enrollees at risk for HIV from January 1, 2011‒December 31, 2013 in the eastern region of the United States. Data extracted from the Truven Health MarketScan database was used to conduct these analyses of HIV testing rates; Truven Health MarketSan is a health insurance claims database compiled from enrollees with employer-sponsored private health insurance including employees, retirees, and dependents. Medicare-eligible retirees with employer-provided Medicare Supplemental plans and Consolidated Omnibus Budget Reconciliation Act (COBRA) enrollees are included in the MarketScan database. Data are representative of the United States’ privately insured population^10^. The database includes paid claims as well as detailed patient information. The specific databases within MarketScan used to obtain the target population for this study were the Commercial Claims and Encounters Database (CCAE); and Medicare Supplemental and Coordination of Benefits Database (MDCR)^10^. The data use agreements only allow data sharing if it is aggregated and fully de-identified as presented in this paper. No interviews were conducted and no informed consent was obtained as human subjects protection procedures were not required. Our research design was reviewed by the CDC Office of Public Health Emergency Preparedness and Response Human Subjects Protection Coordinator and found to be exempt, as this study does not constitute human subjects research, Human Subjects Research #2016083102.

****Enrollment detail data were used to identify enrollees residing in the Hurricane Sandy Impact Area (HSIA). While media coverage focused on impacts in New York and New Jersey, the Federal Emergency Management Agency (FEMA) declared impact area included 21 states: Connecticut, Delaware, Indiana, Illinois, Kentucky, Maine, Maryland, Massachusetts, Michigan, New Hampshire, New Jersey, New York, North Carolina, Ohio, Pennsylvania, Rhode Island, Tennessee, Vermont, Virginia, Washington D.C. and West Virginia. All enrollees residing in the HSIA not identified as having an HIV diagnosis met study eligibility.

**Data Sources and Measurement**

The Federal Emergency Management Agency Modeling Task Force (FEMA MOTF) Hurricane Sandy impact analysis data included storm intensity data (precipitation, buildings lost, households exposed to the surge, etc.) by county. The FEMA MOTF data was compiled by experts in hazard assessment and derived from federal agencies, universities, national labs, state, and local entities. These data were used to develop estimates of impact after the event^11^. The four categories of Final Impact Rank were: a) low, b) moderate, c) high and d) very high. For purposes of this study, SIR was derived by cross-walking the final impact rank assigned to counties to Core Based Statistical Areas (CBSAs) and by calculating a median final impact rank for a given CBSA weighted by the county population estimate for 2012. Rural areas were excluded from this analysis because SIR could not be assigned accurately for combined rural areas in affected states. SIR designated from low to very high was indicated according to the following color scheme: 1) Green (low), 2) Yellow (moderate), 3) Red (high), and 4) Purple (very high) for the CBSAs (see Figure 1).

**Figure:**
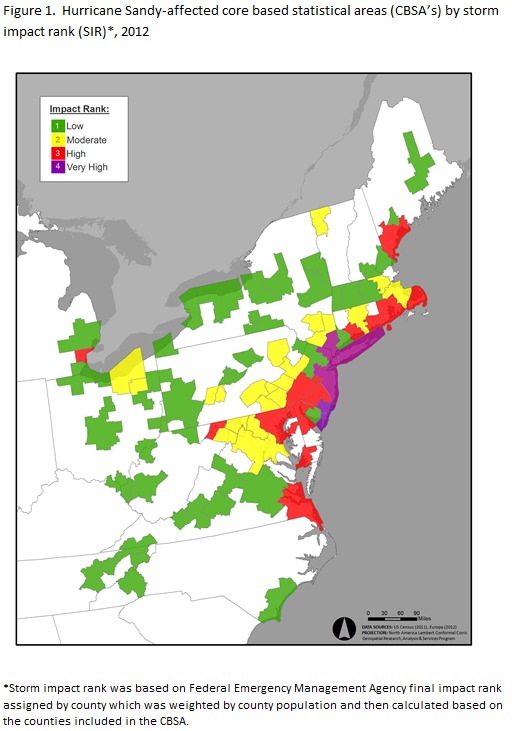

**HIV Testing Rates per 1,000 Enrollees**

HIV testing rates per 1,000 enrollees were calculated as the number of enrollee claims for HIV tests per week divided by total enrollment without previous HIV diagnosis per week and multiplied by 1,000 for all CBSAs in the HSIA. The SIR resulting from analyses of FEMA final impact ranks was linked by CBSA to HIV testing rates. Weekly testing rate time series were then compiled by SIR.

**Statistical Methods**

Descriptive statistics of mean, median, standard deviation, and range were calculated by storm impact rank for HIV testing rates per 1,000 enrollees before and after the event date. There were 153 weeks in each time series with 95 weeks pre-event and 58 weeks post-event. All statistical analyses were conducted using the statistical software package SAS v9.3.

In order to estimate the impact of Hurricane Sandy on HIV testing rates, ITS models of weekly rates were estimated via maximum likelihood for each SIR. Note that we defined the “event” that interrupted the time series as the week of October 29, 2012, the date Hurricane Sandy made landfall in the eastern United States. ITS models were specified as linear segmented regression with autoregressive errors. Details on the methods employed are provided in the Technical Appendix.

ITS models produce estimates of the immediate impact of the storm on HIV testing rates as well as the impact of the storm on the trend in testing rates^12^^, ^13.The parameter estimates produced by the ITS models were used to calculate the percent change in testing rates attributable to the storm and associated confidence intervals immediately and at one, four, eight, and twelve weeks post-event. The estimated absolute change in HIV testing rates attributable to the storm was used to calculate the estimated absolute impact of the event in terms of missed tests, as well as the duration of weeks that testing rates for each SIR was below baseline. Return to baseline was defined as the week that the percent change in testing rate between actual and baseline was no longer statistically significant. (See Technical Appendix)

## Results

Table 1 presents descriptive statistics for weekly HIV testing rates among privately insured enrollees in the HSIA by SIR and the mean HIV testing rates, pre-event (January 1, 2011 ─ October 27, 2012, 95 weeks) and post-event (October 28, 2012 ─ December 31, 2013, 58 weeks). HIV testing rates did not appear to differ greatly when simply considering pre- versus post-event. Figure 2 displays weekly testing rates by SIR during the study period from January 2011‒December 2013. Seasonal variation is evidenced by the periodic fluctuations in weekly testing rates as well as several downward spikes prior to Hurricane Sandy (identified in Figure 2 as a vertical reference line labeled “Event”). A positive relationship between the HSIA and HIV testing rates was apparent prior to the event. The standard deviations (and thereby variance) in testing rates do appear to increase post-event. Sharp downward spikes are observed in Figure 2 occurring at the time of the event. The magnitude of these spikes tend to increase with storm impact rank.

Estimated ITS effects, displayed in Table 2, revealed that HIV testing rates declined significantly in storm-affected areas at each SIR. Relative changes in HIV testing rates were largest immediately following the event, declining towards baseline over time. Similarly, the largest negative effect was observed in the highest SIR immediately following the event (-24%), while the smallest negative effect was observed in the lowest impact rank (-5%). Testing rates returned to baseline in low impact areas by six weeks post-event, December 9, 2012. In moderate impact areas, testing rates returned to baseline by 15 weeks post-event, February 10, 2013. In high and very high impact areas, testing rates returned to baseline by 17 weeks after the event, February 24, 2013. Estimated numbers of missed testing opportunities are displayed in Table 3. By the time testing returned to baseline across SIRs, 9,736 (95% CI: 7,540, 11,925) testing opportunities were missed. The largest amount of missed testing opportunities occurred in very high impact areas, accounting for 6,811 (95% CI: 5,644, 7,978) (see Technical Appendix for detailed results of the ITS regressions).

**Figure:**
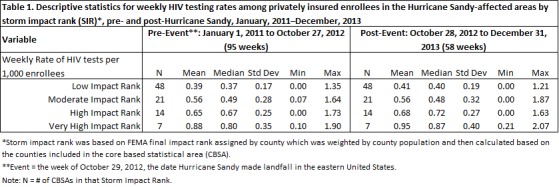

**Figure:**
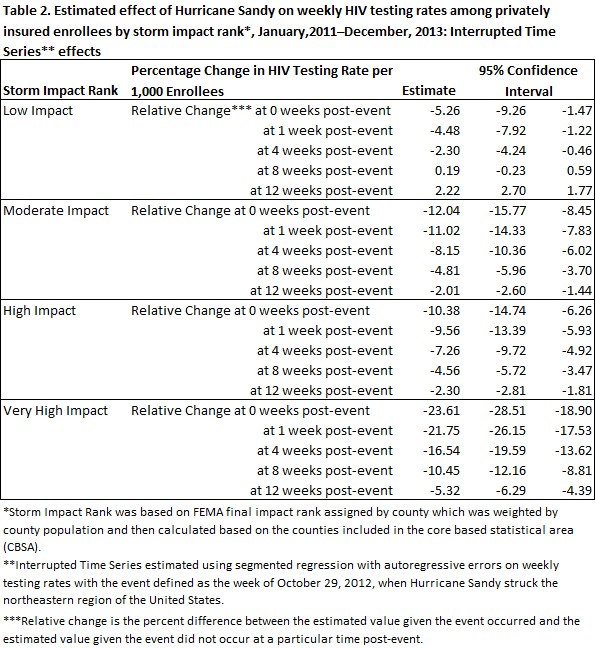

**Figure:**
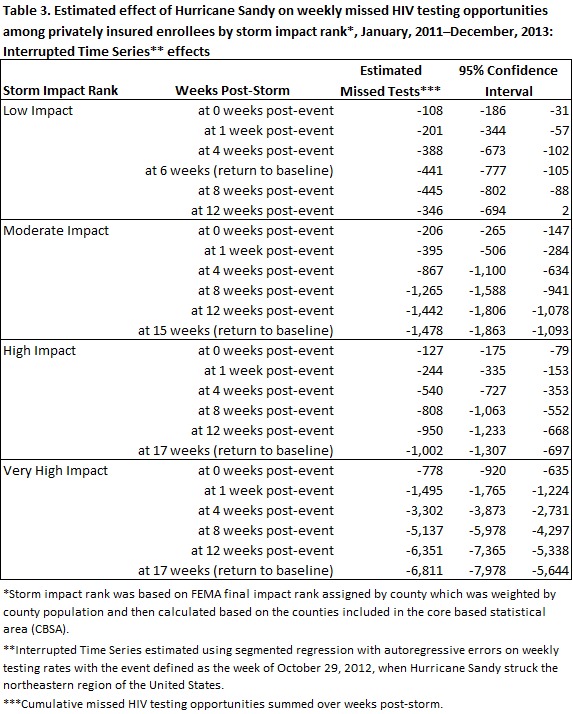

**Figure:**
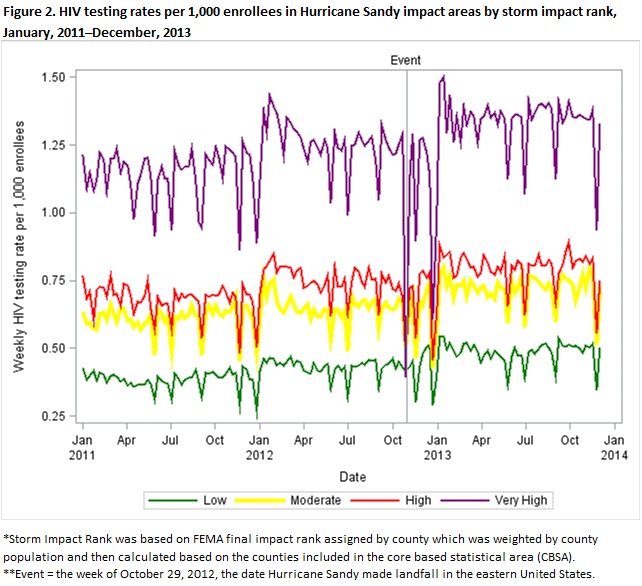

## Discussion

The results of the ITS analysis indicated an immediate decline in HIV testing rates after Hurricane Sandy among privately insured in storm affected areas. Greater disruption in HIV testing was noted in higher impact areas. The relative impact on HIV testing rates was most extreme immediately after the event, returning to baseline over time. Areas with greater storm impact had progressively longer periods of HIV testing disruption. While causality between Hurricane Sandy and declined HIV testing is not established unequivocally, storm impacted areas exhibited declined HIV testing rates and larger declines were associated with higher storm impact rank. We explored the relationship with a time series analysis in order to control for seasonality and autocorrelation and to isolate the independent effect of Hurricane Sandy on HIV testing rates.

Our findings supports earlier studies on the effects of Hurricane Katrina, indicating that areas affected by severe weather events are susceptible to disruption in access to healthcare^14^. Specifically, access to HIV testing and prevention healthcare services was interrupted until five months after the devastation occurred^15^. Severe weather-related disasters often disrupt important health maintenance and health care delivery services. This study found a similar disruptive effect on HIV testing, which became secondary to recovering from the storm and restoring the community.

HIV can persist for up to ten years before symptoms are detected, and when symptoms are revealed, it might be too late to avoid immune system break down and full-blown AIDS. Interruption in availability of HIV testing services could be costly in terms of inadvertent spread of the disease, increased risk of opportunistic infections, late diagnosis, and progression to AIDS among persons unaware of positive status. Shifting from the public health policies of the 1990s and early 2000s, which emphasized evidence based interventions and behavior modification education, the current prevention strategy is a “test and treat” model^16^. The test and treat model of HIV prevention emphasizes identification of HIV-infected persons early in the disease stage, timely initiation of care, and uninterrupted administration of antiretroviral medications to achieve viral load suppression sufficient enough to prevent secondary transmission^16^. Lack of availability of HIV testing eliminates the foundation of this prevention model.

Thus, these results have implications for public health practitioners in high HIV disease burden areas. State and local health jurisdictions should consider assessing the risk of vulnerable populations at risk for HIV infection and working with stakeholder partners to ensure sufficient staff and infrastructure could be maintained in advance of natural disasters to prevent disruptions in services. Moreover, public health practitioners should consider prioritizing restoration of HIV testing as soon as feasible in areas that receive the most damage after an event. In the event of a natural or a man-made disaster, public health and health care organizations serving populations at risk for HIV should have emergency response protocols to educate individuals at risk for HIV^17^. Looking ahead, professionals engaged in preparedness planning for events that might disrupt regular health services, especially those in high HIV burden areas, should be aware of the possibility of HIV testing disruption and have plans in place to avoid long-term gaps in availability of these vital services.

**Strengths and Limitations**

One strength of this study is the use of Interrupted time series (ITS), a robust modeling technique that controls for prior trends and seasonality while estimating the independent impact of an event^12^. ITS specified as segmented regression with autoregressive error is a very strong quasi-experimental research design that produced estimates of immediate as well as extended effects of the event in question^12^^, ^18. While one potential limitation of ITS is confounding by a co-occurring event, the authors did not identify any co-occurring event that might confound these estimated HIV testing rates.

Marketscan is a rich private health insurance claims database that is representative of the privately insured population in the U.S.^10^. Because county identifiers were not available, these analyses were limited to non-rural areas, specifically CBSAs. The storm impact ranks could not be accurately assigned to combined rural areas in each state in the absence of county identifiers. Thus, those who lived in rural areas, were uninsured, or were publically insured were excluded from this study. CBSA-specific effects were also not addressed in this analysis as ITS was conducted by storm impact rank. Therefore, these results might understate the disruption that actually occurred among uninsured, rural, and publically insured populations and ignores CBSA-specific effects.

## Conclusions

Our results suggest that public health preparedness professionals in areas with high HIV disease prevalence should collaborate with HIV testing and treatment providers to enhance readiness in advance of natural or man-made disasters that could disrupt preventative healthcare services. Public health emergency preparedness professionals should consider reviewing their epidemiology data to determine if the populations they serve are at increased risk for HIV infection during an event and work with testing venues and treatment providers to establish testing and treatment provision plans. In high HIV burden areas, an emergency plan for continuity of HIV testing and results services should be a priority. This research supports emergency preparedness in states, local jurisdictions and communities at risk for HIV by highlighting a neglected concern, the potential of HIV testing service interruption during catastrophic events, and suggests advanced planning to resume availability of status determination. These findings can inform public health preparedness policies and practice during a large-scale emergency capable of disrupting normal health care access at the state, local, or community level due to high HIV/AIDS morbidity and disruption of health care services. This research can also inform pre-event planning for disease surveillance in advance of natural disasters to prevent long-lasting disruptions in HIV testing and help prioritize routine healthcare services in areas following a natural or manufactured disaster.

In the Hurricane Sandy impact area, about 70% of the population across all age groups, have private health insurance. Our results are representative of that population, since MarketScan is representative of the privately insured population across all age groups.

Because the approximately 30% of the population that does not have private insurance is typically less healthy^19^, we would expect HIV testing among that population to be more severely impacted. Specifically, HIV testing would experience a sharper decline immediately following Hurricane Sandy and HIV testing would take longer to return to baseline among this (the publically insured and uninsured) population. Future studies would include the effect of Hurricane Sandy on HIV testing rates in the publically insured and uninsured.

## Technical Appendix

**Statistical Methods: Interrupted Time Series**

Claims for HIV tests were extracted from the Truven Health MarketScan database and compiled on a weekly basis for Core-Based Statistical Areas (CBSAs) from January 1, 2011‒December 31, 2013. Enrollees were considered to have a HIV diagnosis if claims were processed on their behalf in the preceding claims year and referenced the following ICD-9-CM codes: ‘042’, ‘0420’, ‘0421’, ‘0422’, ‘0429’, ‘07953’, ‘79571’, and ‘V08’^20^. Thus, enrollees with claims containing HIV diagnoses between January 1, 2010 and December 31, 2010 were excluded from the 2011 database. The remaining enrollees were retained in the reference population for 2011, the population at risk for HIV. Outpatient claims were examined to identify all enrollees without prior HIV diagnosis that had claims for HIV tests ordered using Current Procedural Terminology (CPT) codes (‘86689’, ‘86701’, ‘86703’, ‘87389’, ‘87390’, ‘87534’, and ‘87535’). These CPT codes have been widely used in previously published studies to identify claims for HIV tests performed on privately-insured enrollees to detect HIV and STIs^21^^, ^22^, ^23^, ^24^, ^25.

The ITS was modeled using the following linear segmented regression with autoregressive errors, which represents the baseline level and trend of the outcome variable before the event and changes in the level and trend after the event:

**Figure:**
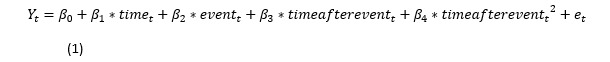

Here, Y_t_ represents the dependent variable at a point in time, weekly HIV testing rates. Time_t_ is the continuous variable representing time in weeks since the beginning of the study period. The intervention function was specified as a step function. Event_t_ is an indicator variable set to zero prior to the date of the event and becoming 1 the week of the event and for the duration of the time series after the event. Timeafterevent_t_ is a continuous variable counting the days elapsed since the event at time t and set to zero prior to the event. The regression error, e_t_, is comprised of a random error component as well as an autoregressive error component to adjust for autocorrelation and seasonality.

The estimated factual case is represented as:

**Figure:**
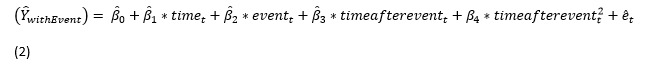

The estimated counterfactual case is represented as:

**Figure:**
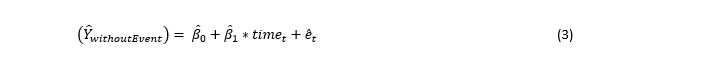

Equation (2) was estimated for each storm impact rank in the Sandy Impact Area. The parameter estimates from equation (2) were used to calculate equation (3) for each storm impact rank.

Note that the parameter estimate β_0_^^^ is the baseline level of the outcome variable at time zero, or the intercept. β_1_^^ ^is the estimated baseline trend of the outcome variable, or the weekly deviation from the baseline level prior to the event. ^^^β_2_ is the estimated absolute change in level, or intercept, of the HIV testing rate that occurs immediately following the event and ^^^β_3_ +^^^β_4_ is the estimated absolute change in trend that occurs after the event. The estimated relative, or percentage, change in the baseline level due to the event is (^^^β_0_ +^^^β_2_ )/^^^β_0_ , while the estimated relative change in trend is (^^^β_1_ +^^^β_3_ +^^^β_4_ )/^^^β_1_ . Thus, ^^^β_0_ +^^^β_2_ is the estimated post-event baseline of the outcome variable and ^^^β_1_ +^^^β_3_ +^^^β_4_ is the estimated post-event trend in the outcome variable. The estimated absolute impact of the event measured one week post-event is equation (2) minus equation (3) evaluated at t=97, or ^^^β_1_ +^^^β_3_ *1+(^^^β_4_ )*1) and the estimated relative impact of the event measured one week post-event is (^^^β_2_ +^^^β_3_ *1+(^^^β_4_ ) *1)/(^^^β_0_ +^^^β_1_ *97).

Thus, absolute impact is the difference between the estimated values given the event occurred and the estimated value given the event did not occur at a particular time post-event. Relative impact is expressed as the percentage change in HIV testing rates in the factual case compared to the counterfactual case. Each time series was tested for stationarity via the augmented Dickey-Fuller test and autocorrelation via generalized Durbin-Watson tests^13^. Autoregressive error models addressed autocorrelation and seasonality. Models were estimated in SAS using “proc autoreg” with autoregressive error terms identified via backward elimination^12^^, ^13^, ^18.

Estimated parameters and standard errors were used to produce estimates and associated 95% confidence intervals (CI) of the relative changes in level and trend as well as the estimated absolute impact of the event in terms of missed tests immediately and at one, four, eight, and twelve weeks post-event as well as at the time that each storm impact rank returned to baseline. Specifically, predicted trends for the factuals and counterfactuals with confidence intervals were retrieved from the regressions for each storm impact rank and used to calculate the relative change and impact at each time point post-event^26^.

## Results: Table and Figure

Table A presents results from interrupted time series regressions by storm impact rank. Parameter estimates from these regressions were used to estimate the predicted factual and counterfactual trends as well as the confidence intervals by storm impact rank that are presented in Figure A. Note that while the estimated impact on the intercept is not always highly significant, the estimated impact on trend is significant for a quadratic trend at p<0.05 across storm impact rank. This produces ITS effects that are significant across storm impact rank.

**Figure:**
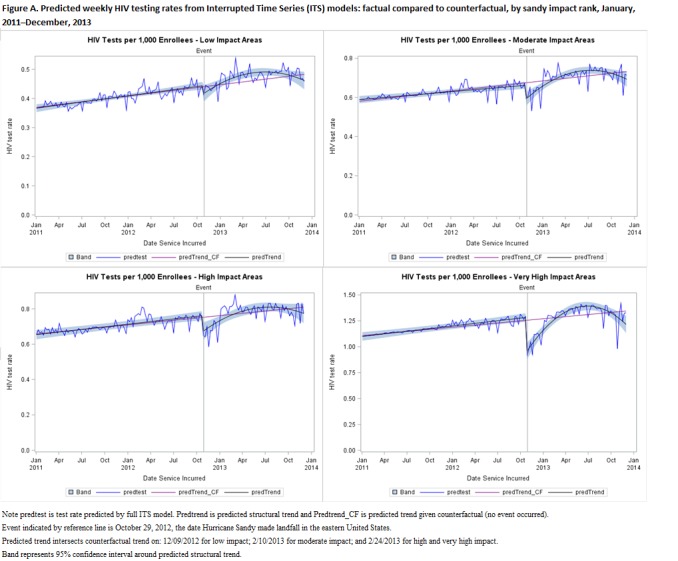

**Figure:**
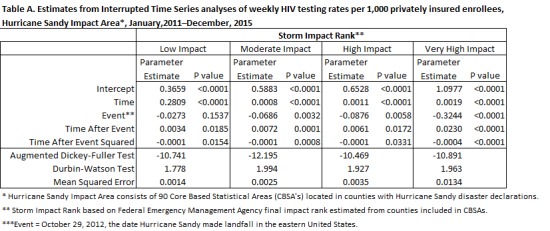

## Data Availability

All relevant data are reported in this manuscript. Data sources include Truven Health Analytics Commercial and Medicare supplemental Claims and Encounters Database, Federal Emergency Management Agency Modeling Task Force (FEMA MOTF) database and Area Health Resource Files (AHRF) data. AHRF and FEMA MOTF data are publicly available and accessible. Data from Truven Health Analytics Commercial and Medicare Supplemental Claims and Encounters Database are designed to address the requirements of the Health Insurance Portability and Accountability Act of 1996 (HIPAA). The MarketScan Research Databases meet the criteria for a limited-use data set and contain none of the data elements prohibited by HIPAA for such data sets. Formal Data Use Agreements (DUAs) are in place with every entity that do not allow public disclosure of detailed datasets used in this study. The DUA’s are in place to protect both patient and hospital sensitive data from public disclosure as the data is classified as a “Limited Data Set” under the Health Insurance Portability and Accountability Act of 1996 (HIPAA) which is to be protected as personal health information (PHI). DUA’s only allow data sharing if it is aggregated and fully de-identified as presented in this paper. Posting detailed data online or providing access to the detailed data that allows replication of this study, or other use of the data to non-public health personnel constitutes a legal violation of these DUA’s.

For requests regarding this data, please contact: Market Scan Information Systems, Inc., 815 Camarillo Springs rd. Suite B, Camarillo, CA. 93012

Phone: 800-MKT-SCAN (658-7226); Fax: 855-MKT-SCAN (658-7226); Email: www.MarketScan.com or www.mDesking.com

## Competing Interests

The authors have declared that no competing interests exist.

## Corresponding Author

Linda I. Ekperi, DrPH, MPH

Applied Science and Evaluation Branch, Division of State and Local Readiness, Centers for Disease Control and Prevention

1600 Clifton Rd., N.E., MS-D44, Atlanta, Ga 30333

Phone: 404-718-6657

Email: wpf7@cdc.gov
